# Photon-Counting CT Material Decomposition in Bone Imaging

**DOI:** 10.3390/jimaging9100209

**Published:** 2023-10-02

**Authors:** Abhisek Bhattarai, Ray Tanaka, Andy Wai Kan Yeung, Varut Vardhanabhuti

**Affiliations:** 1 Department of Diagnostic Radiology, The University of Hong Kong, Hong Kong SAR, China; abhisek89@hotmail.com; 2 Faculty of Dentistry, The University of Hong Kong, Hong Kong SAR, China; rayt3@hku.hk (R.T.); ndyeung@hku.hk (A.W.K.Y.)

**Keywords:** photon-counting detector, material decomposition, computed tomography, bone, osteoporosis

## Abstract

The accurate screening of osteoporosis is important for identifying persons at risk. The diagnosis of bone conditions using dual X-ray absorptiometry is limited to extracting areal bone mineral density (BMD) and fails to provide any structural information. Computed tomography (CT) is excellent for morphological imaging but not ideal for material quantification. Advanced photon-counting detector CT (PCD-CT) possesses high spectral sensitivity and material decomposition capabilities to simultaneously determine qualitative and quantitative information. In this study, we explored the diagnostic utility of PCD-CT to provide high-resolution 3-D imaging of bone microarchitecture and composition for the sensitive diagnosis of bone in untreated and ovariectomized rats. PCD-CT accurately decomposed the calcium content within hydroxyapatite phantoms (*r* = 0.99). MicroCT analysis of tibial bone revealed significant differences in the morphological parameters between the untreated and ovariectomized samples. However, differences in the structural parameters of the mandible between the treatment groups were not observed. BMD determined with microCT and calcium concentration decomposed using PCD-CT differed significantly between the treatment groups in both the tibia and mandible. Quantitative analysis with PCD-CT is sensitive in determining the distribution of calcium and water components in bone and may have utility in the screening and diagnosis of bone conditions such as osteoporosis.

## 1. Introduction

Osteoporosis is a health condition arising from depleting calcium hydroxyapatite contents in bone, compromising bone strength and making it susceptible to breakage. Osteoporosis is a common health concern, especially in elderly and post-menopausal women. Bones undergo constant remodelling by resorption and replacement. However, factors such as age, family history, physical inactivity, small body frame, early menopause, certain medications, etc., can result in bone resorption exceeding reformation and increase the risk of osteoporosis [1]. Hence, assessment of bone health is vital for the diagnosis of osteoporosis and for determining the risk of fracture. Non-invasive imaging modalities, such as magnetic resonance imaging (MRI) and ultrasound, are well-suited for the evaluation of soft tissue. However, their efficacy is limited in appraising dense anatomical elements such as bone. Employing a computed tomography (CT) scanner for bone structure assessment and fracture identification is common. CT imaging offers superb qualitative insights into bone structure, yielding high-resolution images. However, the quantitative assessment of bone quality with a CT scanner alone is not feasible.

With a conventional CT scanner, the mass density of different materials and detected beam energies can result in identical Hounsfield units (HU), hence making the separation between materials difficult. The drawback observed in conventional CT arises from the utilization of energy-integrating detectors (EIDs) for X-ray detection. EIDs operate on multiple layers to convert detected photons into digital signals for image formation. The initial photons are detected in the scintillator layer of the EIDs. The scintillator converts the high-energy incident X-ray photons into photons in the visible spectrum, i.e., low-energy secondary photons. Each of the scintillator layers is separated with septa to prevent light crosstalk between pixels. The low-energy photons in the visible spectrum are absorbed by the photodiode array in the detector unit. The photodiode converts the absorbed photon into an electrical signal in proportion to the total energy deposited in each scintillator unit. The electrical signal is then converted to a digital signal, enabling it to be processed for tomographic image reconstruction. In a specified time, the detector integrates the energy from all incident photons. Hence, information on individual X-ray photons is lost, limiting the capability of EIDs to provide quantitative information.

Current guidelines on bone diagnosis recommend evaluation of patient history followed by dual-energy X-ray absorptiometry (DXA) measurements of bone mineral density (BMD). DXA uses two sets of data based on the attenuation due to high and low-energy X-rays to determine the mass of one material in the presence of another through knowledge of their unique X-ray attenuation at different energies. Two sets of data can be generated by either using two X-ray sources: k-edge imaging or the use of dual-energy detectors. However, DXA resolves three-dimensional material information not as a true volume density but as bone per unit image area (g/cm^2^) in two-dimensional areal density. In addition, DXA measurement fails to provide separate information on cortical and trabecular BMD. Moreover, the trabecular microstructure and voids that influence bone strength also remain undiagnosed [2]. In addition, the metabolically active and varying inner trabecular bone, extraosseous fat distribution, size, structure, and shape contribute to the inaccuracies in measured BMD [3]. The resulting erroneous BMDs have thus led to the misclassification of normal, osteopenic, and osteoporotic bone conditions [4,5].

Multi-material characterization capability in the imaging system can be pivotal in visualizing and quantifying different tissues simultaneously. Fortunately, the multi-material characterization of tissue is now possible with advancements in CT detectors capable of detecting and registering X-rays. Advanced photon-counting detectors (PCDs) allow 3D imaging, quantification, and differentiation between different tissues in the body, such as fat, muscle, and bone. In bone, calcified components in the cortical and trabecular regions may be differentiated from the marrow. The visualization of calcified tissue separately might assist in the accurate assessment of bone’s biomechanical functions. Hence, the ability of high-resolution photon-counting detector CT (PCD-CT) to identify calcified and non-calcified areas in the bone can be pivotal in understanding bone metabolism and the risks of osteoporosis and fracture.

The study of skeletal biology and, in particular, osteoporosis in humans using animal models has been widely accepted. As such, induction of osteopenia with a sex hormone deficiency, simulating human menopause, is common [6,7]. The alveolar ridge and the condyle have been identified in rats as high bone remodelling areas. The effect of oestrogen deficiency is especially pronounced on the trabecular region of the bone, and the degrading effect in the tibia is well documented [8,9]. In the mandible, however, conflicting data persists on the effect of oestrogen deficiency owing to the differences in ovariectomy procedures and the regions of alveolar bone analysis [9,10,11]. The ambiguity in the assessment of osteoporotic changes in the mandible warrants the use of alternative imaging techniques. Therefore, examining the effects of oestrogen deficiency in quantitative and qualitative aspects of bone for diagnosis could be of particular interest. We hypothesize that the spectral sensitivity and material decomposition capability of the PCD-CT scanner could detect minute morphological and compositional changes in the trabecular regions of treated and untreated rat bones.

In addition to quantifying calcium content in bone, determining the distribution of water and adipose tissue in bone marrow is of particular interest. Monitoring soft tissue composition, i.e., the ratio of water to adipose content, can help examine the metabolic activity in bone. Monitoring soft tissue composition by generating virtual non-calcium images has been shown to improve sensitivity, specificity and diagnostic confidence in the exclusion of acute hip fracture over the use of conventional CT reconstruction alone [12]. Virtual non-calcium images have also been generated using dual-energy CT. Photon-counting CT advances on dual-energy CT due to the possibility of the selection of narrower X-ray energy bins, which might lead to the more accurate compositional analysis of tissue. Additionally, the ability to quantify water and fat together or separately improves current diagnostic capabilities. We aim to examine the validity of PCD-CT towards providing high-resolution 3-dimensional images and quantitative information on the distribution of calcified and non-calcified components, which is crucial for determining risks of osteoporosis and fracture, as an alternative to DXA.

## 2. Material and Methods

### Animal Procedures

Eight-week-old Sprague-Dawley rats weighing 230–250 g and free of viral, bacterial and parasitic pathogens were used in the study. The rats were grouped as the ovariectomized group (O-Group, *n* = 8), O+ experimental periodontitis (OP-Group, *n* = 8), sham + EP experimental periodontitis (P-Group, *n* = 8), and sham group (C-Group, *n* = 8). The O- and OP-groups were ovariectomized bilaterally at 12 weeks old, and the other group was subjected to sham surgery. The P- and OP-Groups at 16 weeks underwent dental ligation (Ethicon silk suture 4/0) on the unilateral second maxillary molar to experimentally induce periodontitis. The rats were euthanized at 20 weeks. The samples were prepared for a study with different aims, in which bones on the left side of the rats were utilized. In this study, the tibial and mandibular samples from the right side were used. Immediately after euthanization, the right mandible and tibias were harvested. A total of 2–3 rats of the same group were raised in the same cage under careful daily observation of their physical and mental/psychological conditions for 3 months. 

## 3. Image Acquisition

### 3.1. Photon-Counting Detector CT 

The samples were scanned with a PCD-CT scanner for the quantitative analysis of bone calcium, water and adipose tissue contents and bone structure. The samples were placed on a custom-built plastic sample holder and scanned with a PCD-CT Medipix All-Resolution Scanner (MARS, MARS Bioimaging, Christchurch, New Zealand). The MARS was equipped with a SourceRay SB-120-350 X-ray source (60–120 kVp, 20–350 μA). The samples were scanned with a tube voltage of 120 kV, a tube current of 32 μA, and an exposure time of 200 ms. The scanner had an inherent filtration of 1.8 mm Al equivalent, and an additional filtration of 0.375 mm brass was employed for the scans. The objects were scanned with a field of view of 50 mm, a source-to-object distance of 210 mm, an object-to-detector distance of 65 mm, and a source-to-detector distance of 271 mm.

The key component of the PCD-CT scanner is the state-of-the-art Medipix3RX detector, which is capable of detecting photons and resolving their energies. The interaction of different materials with X-rays varies. The difference in interaction between a material and X-rays is portrayed in its linear attenuation profile. A conventional CT detector employing an energy-integrating detector only utilizes linear attenuation information for image formation. A photon-counting detector, such as Medipix3X, registers the interaction of individual photons and separates them based on energy bins that are pre-defined by the user. The Medipix3X detector can simultaneously operate at four charge-summing counters (CSM) and in three single-pixel modes (SPM) and includes an arbitration counter set at 7 keV. Hence, images are acquired in 8 energy bins simultaneously. The energy deposited in each energy bin is registered and recorded as a separate energy spectrum. Hence, information on the density and atomic makeup of the materials can be used to reconstruct images. The information on atomic makeup and density is processed by a built-in MARS algorithm to determine the materials present in the image. The detector is comprised of a cadmium zinc telluride (CdZnTe) sensor. The quantum detection efficiency of CdZnTe is within the diagnostic energy range of 30–120 keV. The sensor is bump-bonded onto a Medipix3RX complementary metal oxide semiconductor application-specific integrated circuit. The detector is layered with aluminium on the surface and an electronic chip on the base of the detector. Each of the 7 detectors in the system comprises 128 × 128 pixels, and the physical size of each pixel is 110 × 110 μm^2^. 

**Figure 1:**
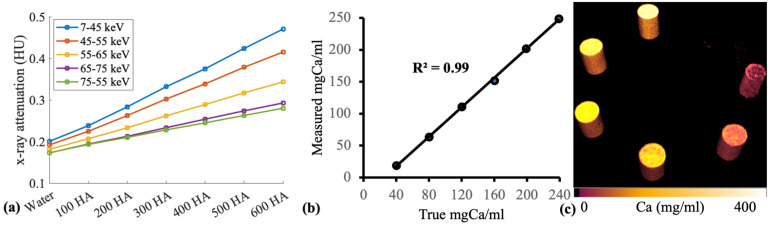
(**a**) Fits of hydroxyapatite phantoms with concentrations of 40 mgCa/mL, 80 mgCa/mL, 120 mgCa/mL, 160 mgCa/mL, 199 mgCa/mL, and 239 mgCa/mL measured at energy bins of 7–45 keV, 45–55 keV, 55–65 keV, 65–75 keV, and 75–120 keV. The plot demonstrates the good spectral resolution of the scanner. (**b**) Fit of true and measured calcium concentrations in hydroxyapatite rods, determined with PCD-CT scanner (MARS, MARS Bioimaging). (**c**) 3D illustration of calcium content in the HA rods placed in order of 40 mgCa/mL, 80 mgCa/mL, 120 mgCa/mL, 160 mgCa/mL, 199 mgCa/mL, and 239 mgCa/mL.

Custom-made hydroxyapatite (HA) phantoms were scanned with PCD-CT. The HA phantoms were composed of calcium in a lipid base. The main objective of this measurement was to establish an optimal imaging protocol with a PCD-CT scanner (MARS, MARS Bioimaging) and assess the image quality and material decomposition capability of the scanner. Six HA phantoms (40, 80, 120, 160, 199, and 239 mgCa/cm^3^) and a plastic vial containing water were scanned (Figure 1).

### 3.2. MicroCT

The samples were also scanned with a microCT scanner (SkyScan 1272, Bruker, Kontich, Belgium). The scan parameters were 100 kV tube voltage, 100 uA tube current, and 3300 ms exposure time. The camera-to-source and object-to-source distances were adjusted to achieve an image pixel size of 12 um. The X-ray beam was filtered with a 0.11 mm Cu filter to lower the beam-hardening effect. Two hydroxyapatite phantoms, 0.25 and 0.75 gcm^−3^, were placed along with the specimens during the scan to facilitate the quantification of bone mineral density. The use of HA phantoms in the scans enabled the determination of calcium contents in the bone samples. The linear attenuation coefficient of the phantoms was employed to convert the X-ray attenuation information of the bone in Hounsfield units into quantitative material information (mgCa/mL).

### 3.3. Image Analysis

Analyses of morphological bone parameters and concentration of calcified and non-calcified tissue in the alveolar region below the second molar and at the proximal tibia were performed [13]. The microCT and MARS datasets were analyzed with the image processing and analysis tools Nrecon (Bruker, Belgium) and Visions (MARS Bioimaging, Christchurch, New Zealand), respectively. For the PCD-CT scan, the region of interest in the alveolar region and proximal tibia was manually selected using the Visions software. Based on the manually selected data points, material information throughout the selected volume was generated by Visions. Similarly, Nrecon software was employed to semi-automatically define the region of interest. The attenuation data in HU was then converted into material information. 

## 4. Statistical Analysis

All the statistical analyses were performed with SPSS (IBM SPSS Statistics for Windows, Version 27.0. Armonk, NY, USA). Shapiro–Wilk tests revealed the scan data followed a normal distribution. The samples were divided into ovariectomized and control groups. One-way ANOVA was used to examine the statistical difference between the control and the ovariectomized groups. The statistical significance of tests was set at *ρ* < 0.05. 

## 5. Results

**Figure 2:**
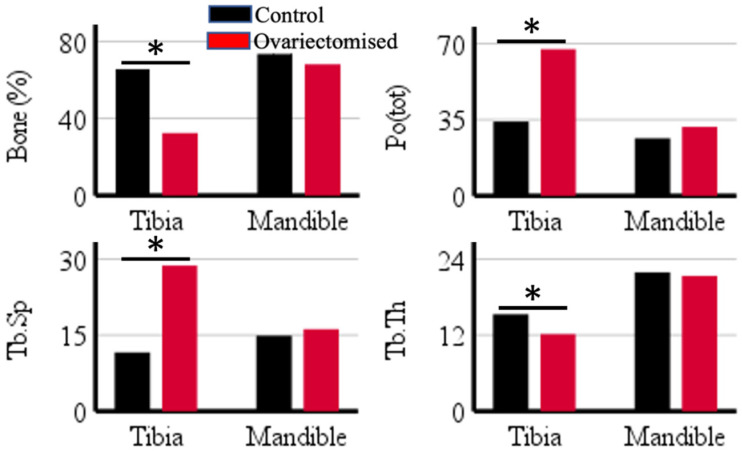
The mean difference in morphological parameters (bone percentage, trabecular thickness (Tb.Th), trabecular separation (Tb.Sp), total porosity (Po(tot)) between control and ovariectomized group in tibia and mandible. A significant difference between the mean values is represented in the figure with *. The statistical significance of tests was set at *p* < 0.05.

The PCD-CT scanner accurately quantified calcium content in hydroxyapatite phantoms with concentrations of 40, 80, 120, 160, 199, and 239 mgCa/mL (*r* = 0.99) (Figure 1). Ovariectomy resulted in significant changes in bone percentage (*ρ <* 0.001), total porosity (*ρ* < 0.001), trabecular thickness (*ρ* = 0.004), and trabecular separation (*ρ* < 0.001) when compared to the untreated samples in the tibia. However, a difference in morphological parameters was not observed between the treated and untreated groups in the alveolar bone (Figure 2). 

**Figure 3:**
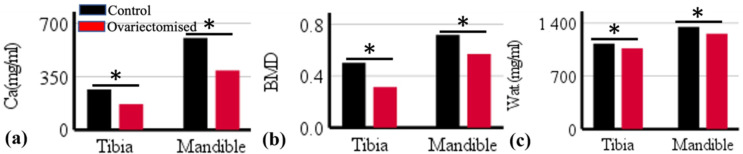
(**a**) Calcium and (**c**) water content quantified with photon-counting CT scanner and (**b**) bone mineral density quantified using microCT dataset in rat tibia and mandible. PCD-CT revealed a significant difference in the calcium and water content of bone in treated samples when compared to the untreated group. A significant difference between the mean values is represented in the figure with *. The statistical significance of tests was set at *p* < 0.05.

Bone mineral density quantified using microCT imaging correlated significantly with calcium content decomposed by PCD-CT (*r* = 0.82) (Figure 3b). Water and calcium content in bone correlated significantly (*r* = 0.88). PCD-CT determined calcium concentration in control and ovariectomized samples and revealed significant differences in the tibia (*ρ* = 0.000) and mandible (*ρ* = 0.004) (Figure 3). Similarly, a significant difference in bone mineral density was observed between the control and treated samples in both the tibia (*ρ* = 0.004) and mandible (*ρ* = 0.003).

## 6. Discussion

This study has demonstrated that PCD-CT can be effective for the characterisation of bone conditions. The spectral sensitivity and material decomposition capability of PCD-CT was tested with hydroxyapatite phantoms (*n* = 6) with varying calcium concentrations ranging from 40 to 239 mg/mL at selected energy bins of 7–45, 45–55, 55–65, 65–75, and 75 kV. Spectral data revealed clear separation in X-ray attenuation between phantoms in separate energy bins (Figure 1a). The optimal separation of X-ray attenuation in different energy bins is particularly useful in obtaining accurate material-specific information. PCD-CT measured calcium content within hydroxyapatite phantoms with high accuracy (*r* = 0.99) (Figure 1b). In addition, the 3-D calcium map of the phantoms also allowed the assessment of the calcium distribution within each rod (Figure 1c).

Phantom measurements were followed by examining the potential of PCD-CT in the diagnosis of bone conditions. As such, untreated rats and ovariectomized ones provided an excellent sample set for examining the scanner’s diagnostic potential. In this study, microCT imaging was utilized for the assessment of morphological parameters, such as bone percentage, total porosity, trabecular thickness, and trabecular separation. Ovariectomy had a deteriorating effect on the trabecular bone in the tibia, as revealed by a significant difference in the morphological parameters when compared to the controls (*ρ* < 0.05). This result corroborates with reports on the deficiency of ovarian hormone in inducing bone loss [14].

The structural assessment of the effects of ovariectomy on the alveolar bone of the treated groups showed that it did not differ significantly from the untreated ones. Ovariectomy was performed on 12-week-old rats, a suitable age for the procedure, as the maturation of skeletal growth in rats is comparable to the bone density and structure of a human skeleton. Any ovariectomy before 12 weeks or 3 months would undergo competing skeletal growth and dwarf any ovariectomy-induced changes. Hence, changes in alveolar bone condition in ovariectomized rats after 12 weeks have been reported, and changes were expected in this study [7] despite conflicting data on the changes in the mandible after inducing estrogen deficiency [11]. The rats were euthanized at 20 weeks, i.e., 8 weeks after ovariectomy, and the timespan might have been insufficient for the hormonal deficiency to have a significant effect on bone morphology [7]. Interpretation of morphological parameters in the mandible is complicated and has been reported to be affected by the length of ovariectomy delay and region of bone analysis [10,11].

**Figure 4:**
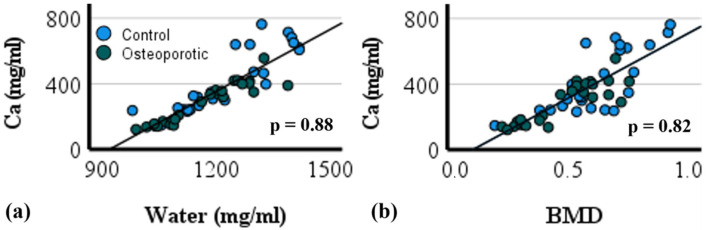
(**a**) Correlation between calcium and water composition in the trabecular and alveolar region of tibia and mandible. (**b**) Fit of material decomposition (calcium) with PCD-CT and bone mineral density.

In addition to morphological characterization, quantitative information on bone quality based on BMD offers a different dimension to the study of bone health. Bone mineral density was quantified after scanning bone samples simultaneously with hydroxyapatite rods using a microCT scanner. In the tibia, ovariectomized and untreated groups differed (*ρ* < 0.05) in bone mineral density (BMD). Similarly, in the alveolar bone of the mandible, a significant difference in BMD was observed between the treated and control groups. The calcium concentration decomposed using PCD-CT correlated significantly with BMD quantified with the use of microCT scanning (*r* = 0.82) (Figure 4). 

**Figure 5:**
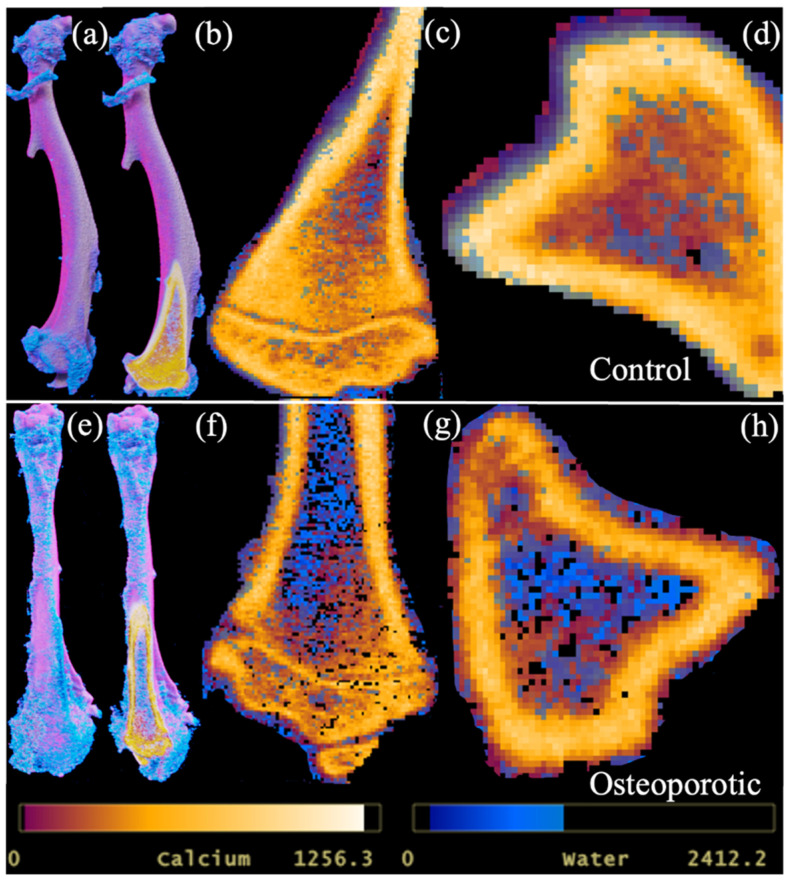
Calcium and water map of the tibia in control (**a**–**d**), and ovariectomized sample (**e**–**h**). A 3-D view of calcium and water map in a control sample (**a**,**b**). Sagittal (**c**) and axial (**d**) view of the inner trabeculae surrounded by dense cortical bone. Similarly, ovariectomized (**e**,**f**) samples in 3-D view. Water and calcium content in the trabeculae surrounded by dense calcium content in sagittal (**g**) and axial (**h**) views in the ovariectomized sample. Calcium content is low and free water content is high in the ovariectomized sample when compared to the control.

Due to calcium being a major constituent, its significant correlation with bone mineral density could be expected. More importantly, PCD-CT demonstrated its ability to provide quantitative information on bone conditions and allowed the three-dimensional visualization of the distribution of calcium and water components in bone (Figure 5). 

PCD-CT quantified differences in tibial and mandibular calcium contents in the trabeculae and alveolar regions as differing significantly between the control and ovariectomized groups. A similar difference in bone mineral density determined with microCT was observed between the groups. However, morphological parameters in the mandible showed no differences between the ovariectomized and untreated groups, as revealed by quantitative information on calcium and bone mineral content. 

The quantitative analysis of CT images has demonstrated greater sensitivity and higher accuracy in diagnosis compared to qualitative assessment [15]. Qualitative data generated with PCD-CT utilizes multiple X-ray energy bins, preserving discrete spectral information [16,17]. In conventional energy-integrating detectors, discrete spectral information is lost, reducing sensitivity in qualitative as well as quantitative information [15,18]. In particular, the quantitative analysis of multi-material components is affected by the loss of discrete data [18]. Material composition, mass density, and photon energy all contribute to image formation, while the linear attenuation coefficient µ(E) is not material-specific nor unique. Hence, the accurate quantification of multiple materials is possible with PCD-CT as the numbers of photons and their energy are registered. Hence, the material decomposition with PCD-CT is sensitive for the accurate detection of multiple components and is useful for diagnosing bone health.

Material decomposition with PCD-CT is not only limited to quantifying hard calcified tissue but can simultaneously detect non-calcified components in bone, such as water and adipose tissue. Adipose tissue is prevalent in bone trabeculae, which is an important indicator of bone metabolism [19]. Osteoporotic changes in bone are accompanied by a loss in calcified tissue and an increase in adipose tissue. The PCD-CT scan protocol was designed to assess and characterize lipids in addition to water and calcium in bone. PCD-CT determined a significant difference in water content between the ovariectomized and untreated groups. Water content in bone influences its mechanical strength and determines its fracture resistance by providing ductility [20]. Bound water percentage decreases and pore water content in bone increases with age [20]. Ageing is also associated with a decrease in bone mineralization and loss of strength and ductility. The results from this study corroborate with reports on the changes in bound and free water content in treated and untreated bones (Figure 3 and Figure 5). The measurement of lipid content within the HA phantoms was possible. However, the distribution of lipids within the excised rat tibia and mandible was not detected. The fixative solution could have caused the dissolution of lipid content within the samples. In future studies, the use of fresh bone samples may allow the detection of the major components within the bone.

## 7. Conclusions

PCD-CT demonstrated its effectiveness in the qualitative and quantitative diagnosis of bone conditions. Moreover, the spectral sensitivity of PCD-CT was effective in detecting subtle changes in calcium and water contents between the control and treated tibial and mandibular bones. PCD-CT can be an improved alternative for the diagnosis of bone conditions, monitoring treatment, and assessing the risk of fracture. 

## Data Availability

The study’s data is available upon reasonable request.
